# New Orleans Medical Journal

**Published:** 1849-10

**Authors:** 


### NEW ORLEANS MEDICAL JOURNAL.

This valuable journal appears in a new dress, having entered upon the sixth volume. We refer to it now for the purpose of recommending it to our readers resident in the South as a journal especially suited to their wants. At every stage of its existence it has been conducted with judgment and ability, and claims one of the best corps of contributors in the country. It is now under the sole guardianship of Dr. Hester.

